# Difference in fatigue and pain between neuromyelitis optica spectrum disorder and multiple sclerosis

**DOI:** 10.1371/journal.pone.0224419

**Published:** 2020-04-06

**Authors:** Hiroki Masuda, Masahiro Mori, Akiyuki Uzawa, Tomohiko Uchida, Ryohei Ohtani, Satoshi Kuwabara

**Affiliations:** Department of Neurology, Graduate School of Medicine, Chiba University, Chuo-ku, Chiba, Japan; Charite Universitatsmedizin Berlin, GERMANY

## Abstract

**Objective:**

To investigate the difference of fatigue and pain in patients with neuromyelitis optica spectrum disorder (NMOSD) and multiple sclerosis (MS).

**Methods:**

Data from the Modified Fatigue Impact Scale (MFIS) and Pain Effects Scale (PES) were compared between 51 NMOSD and 85 MS patients. Each score was compared in each disease group with or without clinical abnormalities. Since almost no MS patients are without brain magnetic resonance imaging abnormalities, volumetry analysis by the Lesion Segmentation Tool and statistical parametric mapping 12 were added to obtain total lesion volume and intracranial volume in MS patients, and the correlations between total lesion volume/intracranial volume and each score were investigated.

**Results:**

Compared to the MS group, the NMOSD group showed a higher PES score (median, 15.0 vs. 7.0, *P* = 0.045), no difference in MFIS, and an increased percentage of patients with extended spinal cord lesions (58.8% vs. 8.2%, *P* < 0.001). Moreover, NMOSD and MS patients with extended spinal cord lesions tended to demonstrate higher PES scores than those without. A positive correlation between MFIS and PES were found in patients with NMOSD and MS. On the other hand, MS patients showed a higher percentage of brain abnormalities (80.4% vs. 97.6%, *P* = 0.001) and a positive correlation between total lesion volume/intracranial volume and MFIS (Spearman’s ρ = 0.50, *P* = 0.033).

**Conclusions:**

The origin of fatigue may be associated with spinal cord lesions causing pain in NMOSD patients, but with brain lesions in MS patients.

## Introduction

Neuromyelitis optica spectrum disorder (NMOSD) is a severe inflammatory disease in the central nervous system mostly affecting the optic nerve and the spinal cord [1]. One feature of NMOSD is the positivity of anti-aquaporin-4 antibodies [2]. Recently, several NMOSD patients were reported to have brain lesions [3]. On the other hand, multiple sclerosis (MS) is a demyelinating disorder in the central nervous system with features of dissemination of demyelinating lesions in time and space [4].

Many factors, including pain, depression, cytokine and endocrine factors, and poor sleep, can cause fatigue in patients with neurological diseases [5, 6]. It was reported that the primary fatigue in patients with MS may stem from centrally mediated processes caused by the conduction issues such as demyelination or axonal loss in CNS [7]. In NMOSD and MS, fatigue has been reported to impair activities of the daily living [6, 8–10]. In MS patients, the prevalence of fatigue was reported to be 50% to 90%, and more than 20 trials were performed to determine the appropriate treatment for fatigue [6, 11]. Another study showed 36.5% of early MS patients exhibited fatigue [12]. Subjective feeling of fatigue was reported to be significantly decreased by group support, individual cognitive behavioral interventions, and a professionally guided self-care management program [7, 13, 14]. A previous study reported a similar incidence of fatigue in NMOSD and MS patients, with a strong correlation between depression and fatigue [9]. On the other hand, chronic pain is a common symptom causing deterioration of activities of the daily living in patients with MS and NMOSD [15–17]. However, the relationship between fatigue and pain has not been fully investigated, particularly in NMOSD patients.

We investigated the difference in fatigue and pain in patients with NMOSD and MS.

## Methods

### Standard protocol approvals and patient consents

The study procedure was approved by the ethics committee of the Chiba University School of Medicine (No. 1937). All patients provided informed consent.

### Study design and patient populations

The Modified Fatigue Impact Scale (MFIS), the Multidimensional Fatigue Inventory (MFI), and the Pain Effects Scale (PES) were distributed to 77 consecutive patients with NMOSD and 221 consecutive patients with relapsing-remitting MS at the Chiba University Hospital. All patients were requested to answer all questions. Patients who answered to all of the three scales including MFIS, MFI, and PES without blanks were enrolled in this study. We added the retrospective analysis to those people. In total, 51 NMOSD and 85 MS patients answered the MFIS, MFI, and PES without blanks. All patients with NMOSD fulfilled the 2015 International consensus diagnostic criteria [1], while the MS patients fulfilled the 2010 McDonald’s diagnostic criteria [4]. Patients with NMOSD or MS that relapsed within 1 month of these tests and those who were unable to perform any test due to cognitive dysfunction, severe bilateral visual loss, or disturbance in the dominant upper extremities (Medical Research Council grade ≤ 3) were excluded.

Demographic and clinical features, including sex, age at MFIS administration, disease duration, duration from last attack to MFIS administration (in months), Kurtzke Expanded Disability Status Scale (EDSS) score at MFIS administration, presence of residual disability (visual acuity < 0.1 or a Medical Research Council manual muscle testing score < 3), and number of patients with a history of optic neuritis (ON) and myelitis and treatment at MFIS administration were reviewed. These parameters were obtained during the study. Patients with anti-aquaporin-4 antibodies positivity, brain magnetic resonance imaging (MRI) abnormalities, and the past history of the extended spinal cord lesions (>3 vertebral segments) were also investigated. Brain MRI follow-up is regularly performed about once a year in patients with NMOSD and MS. The brain MRI abnormality was assessed by the last brain MRI performed before MFIS/MFI/PES. The past history of the extended spinal cord lesions was obtained from the medical record in the acute phase of myelitis. Each patient regularly takes brain MRI follow-up. Anti-aquaporin-4 antibody levels were measured as reported previously [18].

MFIS, MFI, and PES scores were compared between patients with and without items, including history of ON, visual acuity fixed at <0.1, history of myelitis, extended spinal cord lesions, and brain MRI abnormalities, to investigate the differences in fatigue features between NMOSD and MS patients. However, the number of MS patients without MRI abnormalities was expected to be extremely low; therefore, brain volume measurements were added to compare the brain lesion volume and fatigue or pain scores in these patients.

### Brain volume measurements

We compared the correlations between brain lesions and scores, including MFIS, MFI, and PES, in patients with MS using statistical parametric mapping (SPM) 12 implemented on the Matlab version R2016b for Windows 10 (MathWorks, Inc., Natick, MA, USA). Intracranial volume (ICV), calculated by the sum of the whole brain gray matter, white matter, and cerebrospinal fluid volumes, was treated to normalize for head size. Lesions were segmented by the lesion growth algorithm as implemented in the Lesion Segmentation Tool (LST) toolbox version 2.0.15 (www.statisticalmodelling.de/lst.html) for SPM [19]. We used the initial threshold (κ) value of 0.30 as the investigator recommended [19]. Lesion volume filled by LST was expressed as total lesion volume (TLV). Brain volume was measured only to MS patients whose MRI was performed within 60 days before MFIS.

### Brain MRI in the correlation study

MS patients who underwent brain MRI, including conventional brain MRI, T1-weighted three-dimensional images, and fluid-attenuated inversion recovery (FLAIR) within two months before MFIS, were included in the correlation study. Five MRI systems were used in this study: 1.5-Tesla Signa HDxT (GE Healthcare, Milwaukee, WI, USA), 3.0-Tesla Discovery MR 750 (GE Healthcare), 1.5-Tesla Achieva (Philips, Amsterdam, The Netherlands), 1.5-Tesla Achieva dStream (Philips), and 3.0-Tesla Ingenia (Philips) scanners. Details of MRI systems are shown in Table 1.

**Table 1:** Details of MRI systems.

	Signa HDxT	Discovery MR 750	Achieva	Achieva dStream	Ingenia
Number of patients	7	6	3	1	1
Tesla	1.5	3.0	1.5	1.5	3.0
**3DT1**					
FOV (mm×mm)	240 × 240	220 × 220 to 256 × 256	230 × 230	240 × 240	240 × 240
Number of sections	248	178 to 248	127 to 128	256	260
Section thickness (mm)	1.4	1.0 to 1.4	1.5	1.4	1.4
TR (ms)	7	7 to 8	22	22	8
TE (ms)	2.9 to 3.0	3.0 to 3.3	4.6	4.6	4
TI (ms)	0 to 420	420	NA	NA	NA
Number of signals acquired	0.9921 to 1	0.5 to 1	1	1	1
Echo train length	1	1	1	1	240
FA	15°	15°	25°	25°	8°
**FLAIR**					
FOV (mm×mm)	220 × 220	220 × 220	220 × 220	220 × 220	220 × 220
Number of sections	32	32	32	32	32
Section thickness (mm)	4	4	4	4	4
TR (ms)	10000 to 10002	10000	11000	11000	10000
TE (ms)	120.2 to 128.6	131.2 to 131.8	120	120	120
TI (ms)	2400	2336.49 to 2500	2800	NA	2700
Number of signals acquired	1	1	2	1	1
Echo train length	1	1	38	38	28
FA	90°	90° to 111°	90°	90°	90°

3DT1weighted three-dimensional images; FA: flip angle; FLAIR: fluid attenuated inversion recovery; FOV: field of view; NA: not acquired; TR: repetition time; TE: echo time; TI: inversion time.

### Statistical analysis

Continuous data were compared between the test and control groups using the Mann–Whitney *U* test. Categorical outcomes were evaluated using the chi-squared test. The Spearman’s rank test was performed to analyze correlations. Analysis of covariance (ANCOVA) was used when MFIS/MFI/PES were compared between NMOSD and MS patients. *P* < 0.05 was considered statistically significant. Statistical tests were conducted using the SPSS version 25.0 (IBM Corporation, Armonk, NY, USA). Due to the exploratory nature of the study no adjustment for multiple comparisons was made.

## Results

### Demographics, clinical characteristics, laboratory findings and treatments of patients with NMOSD and MS

Table 2 shows the demographics, clinical characteristics, and laboratory findings in patients with NMOSD and MS. Approximately 90% of the NMOSD and 77.6% of the MS patients were female. The median age of the NMOSD patients at the time of MFIS/MFI/PES was higher than that of the MS patients (52.0 vs. 42.0 years; range, 26–78 vs. 17–73, *P* < 0.001). The disease duration at MFIS/MFI/PES was lower (median, 8.0 vs. 11.0; range, 0–36 vs. 0–44; *P* = 0.019), the number of months from last attack to MFIS/MFI/PES was lower (median, 24.0 vs. 37.0; range, 1–104 vs. 1–400; *P* = 0.007), the EDSS at MFIS/MFI/PES was higher (median, 3.0 vs. 2.5; range, 0–8.5 vs. 0–8.0; *P* = 0.004), and the percentage of patients with visual acuity fixed at <0.1 and with a past history of ON was higher (25.5% vs. 1.2%, *P* < 0.001; 70.6% vs. 40.0%, *P* = 0.001) in NMOSD than in MS patients. Although the number of patients with history of myelitis was lower among NMOSD patients (84.1% vs. 97.6%, *P* = 0.006), the number of patients with extended spinal cord lesions was higher than that among MS patients (58.8% vs. 8.2%, *P* < 0.001). On the other hand, fewer NMOSD patients had brain MRI abnormalities than MS patients (80.4% vs. 97.6%, *P* = 0.001). Of the NMOSD patients, 48 received immunomodulating treatment, including continuous oral prednisolone (*n* = 47), azathioprine (*n* = 9), and tacrolimus (*n* = 1), while 57 MS patients received disease-modifying drugs, including interferon β-1a (*n* = 18), interferon β-1b (*n* = 12), fingolimod (*n* = 26), and dimethyl fumarate (*n* = 1). One MS patient received continuous oral prednisolone due to coexistent chronic inflammatory demyelinating polyneuropathy.

**Table 2:** Demographic, clinical characteristics, laboratory findings and treatment in NMOSD and MS patients.

	NMOSD (n = 51)	MS (n = 85)	*P-value*
**Demographic and clinical features**			
Female (%)	46/51 (90.2%)	66/85 (77.6%)	*0*.*068*
Age at MFIS/MFI/PES (years)	52.0 [18.0]	42.0 [13.0]	*< 0*.001*
Disease duration (years)	8.0 [13.0]	11.0 [10.0]	*0*.019*
Months from last attack to MFIS/MFI/PES	24.0 [49.0]	37.0 [56.0]	*0*.007*
EDSS score at MFIS/MFI/PES	3.0 [4.0]	2.5 [3.0]	*0*.004*
Visual acuity fixed at < 0.1 in at least one eye	13/51 (25.5%)	1/85 (1.2%)	*< 0*.001*
MRC grade fixed at < 3 in at least one limb	6/51 (11.8%)	2/85 (2.4%)	*0*.*052*
Number of patients with a history of ON	36/51 (70.6%)	34/85 (40.0%)	*0*.001*
Number of patients with a history of myelitis	43/51 (84.1%)	83/85 (97.6%)	*0*.006*
**Laboratory findings**			
Positive anti-aquaporin-4 antibodies	46/51 (90.2%)	0/89 (0.0%)	*< 0*.001*
Patients with brain MRI abnormalities	41/51 (80.4%)	83/85 (97.6%)	*0*.001*
Patients with extended spinal cord lesion**	30/51 (58.8%)	7/85 (8.2%)	*< 0*.001*

Data are presented as median [interquartile range] or number (%).

*P < 0.05. EDSS: Kurtzke’s Expanded Disability Status Scale; MFI: multidimensional fatigue inventory; MFIS: modified fatigue impact scale; MRC: Medical Research Council; MS: multiple sclerosis; NMOSD: neuromyelitis optica spectrum disorder; ON: optic neuritis; PES: pain effects scale.

** Patients with extended spinal cord lesion: patients who showed spinal cord lesion more than three vertebral segments in the acute phase of myelitis.

### MFIS, MFI, and PES scores in NMOSD and MS patients

The MFIS, MFI, and PES scores in NMOSD and MS patients are shown in Table 3. Only PES was higher in NMOSD than in MS patients (median, 14.0 vs. 12.0; interquartile ratio [IQR], 12.0 vs. 10.0; range, 6–30 vs. 6–28; *P* = 0.009). No differences were found in MFIS and MFI between NMOSD and MS patients (median, 42.0 vs. 35.0 and 59.0 vs. 59.0; IQR, 29.0 vs. 28.0 and 28.0 vs. 20.0; range, 0–77 vs. 0–78 and 20–91 vs. 20–91; *P* = 0.43 and 0.19, respectively). All three MFIS subscales, including physical, cognitive, and psychosocial subscales, also showed no differences between NMOSD and MS patients (*P* = 0.071, 0.62, and 0.16, respectively).

**Table 3:** Results of MFIS, MFI, and PES in patients with NMOSD and MS.

	NMOSD (n = 51)	MS (n = 85)	*P-value*
**MFIS**			
physical subscale	21.0 [18.0] (0–36)	16.0 [16.0] (0–36)	*0*.*071*
cognitive subscale	13.0 [11.0] (0–36)	16.0 [11.5] (0–40)	*0*.*62*
psychosocial subscale	4.0 [4.0] (0–8)	3.0 [4.0] (0–8)	*0*.*16*
total score	42.0 [29.0] (0–77)	35.0 [28.0] (0–78)	*0*.*43*
**MFI**			
total score	59.0 [28.0] (20–91)	59.0 [20.0] (20–91)	*0*.*19*
**PES**			
total score	14.0 [12.0] (6–30)	12.0 [10.0] (6–28)	*0*.009*

Data are presented as median [interquartile range] (range).

*P < 0.05. MFIS: modified fatigue impact scale; MFI: multidimensional fatigue inventory; MS: multiple sclerosis; NMOSD: neuromyelitis optica spectrum disorder; PES: pain effects scale.

### Correlations among test scores in patients with NMOSD and MS

MFIS total score showed a positive correlation with MFI and PES in NMOSD (Spearman’s ρ = 0.51 and 0.52, respectively, *P* < 0.001 for both comparisons) and MS (Spearman’s ρ = 0.67 and 0.73, respectively, *P* < 0.001 for both comparisons) patients.

### MFIS, MFI, and PES scores in NMOSD and MS patients with or without a history of ON or with or without visual acuity fixed at <0.1

No differences were found in MFIS, MFI, and PES with or without a history of ON or with or without visual acuity fixed at <0.1 in NMOSD (*P* > 0.29) or MS (*P* > 0.14) patients.

### MFIS, MFI, and PES scores in NMOSD and MS patients with or without a history of myelitis or with or without extended spinal cord lesions

**Fig 1:**
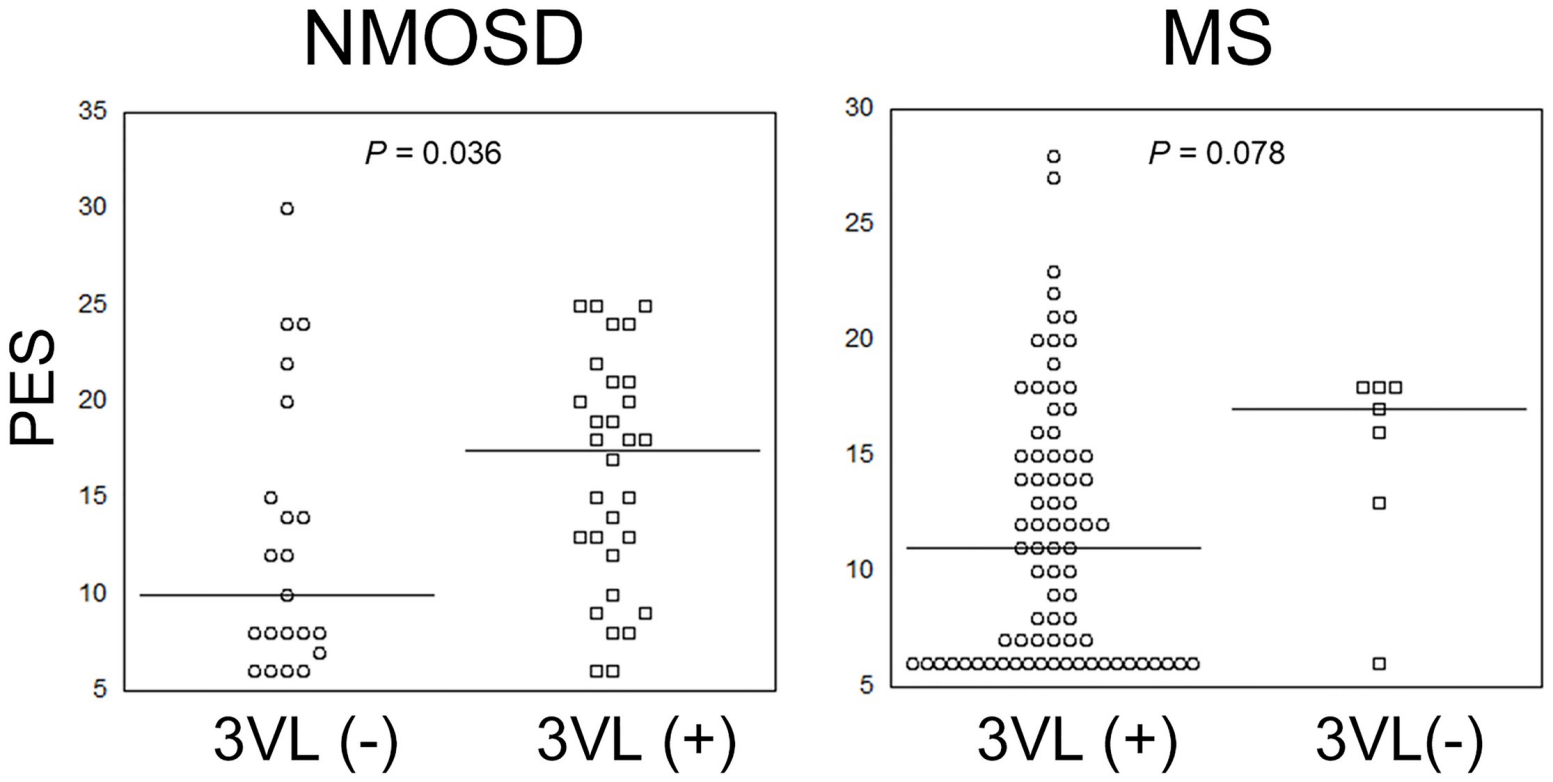
Comparison of PES scores in NMOSD and MS patients with or without extended spinal cord lesions (>3 vertebral segments). Black lines show the medians of each group. MS, multiple sclerosis; NMOSD, neuromyelitis spectrum disorder; PES, Pain Effects Scale; 3VL, spinal cord lesion >3 vertebral segments.

In NMOSD patients, PES was higher in patients with than in those without a history of myelitis (median, 15.0 vs. 7.0; IQR, 12.0 vs. 8.8; *P* = 0.045), and in patients with than in those without extended spinal cord lesions (median, 17.5 vs. 10.0; IQR, 9.5 vs. 10.0; *P* = 0.036). MFIS and MFI showed no differences in NMOSD patients with or without a history of myelitis or extended spinal cord lesions (*P* > 0.17). On the other hand, PES scores tended to be higher in MS patients with than in those without extended spinal cord lesions (median, 17.0 vs. 11.0; IQR, 5.0 vs. 9.0; *P* = 0.078). There were no differences in MFIS, MFI, and PES scores between MS patients with and without a history of myelitis (*P* = 0.23, 0.12, and 0.47, respectively), or in MFIS and MFI scores between MS patients with and without extended spinal cord lesions (*P* = 0.28 and 0.20, respectively). PES scores in all patients with or without extended spinal cord lesions are shown in Fig 1. Disease duration was not different between NMOSD patients with the past history of myelitis and without it (median, 9.0 vs 4.0; IQR, 13.0 vs 3.5; *P* = 0.18).

### MFIS, MFI, and PES scores in NMOSD and MS patients with or without brain MRI abnormalities

No differences were noted in MFIS, MFI, and PES scores between NMOSD patients with and without brain abnormalities (*P* = 0.17, 0.17, and 0.18, respectively). However, the physical subscale in MFI tended to be higher in NMOSD patients with brain abnormalities (median, 24.0 vs. 10.5; IQR, 16.5 vs. 14.0; *P* = 0.077). No difference in MFIS, including subscales, MFI, or PES was found between MS patients with or without brain abnormalities (*P* > 0.11). No differences were found in the disease duration between MS patients with brain MRI abnormalities and without them (median, 15.0 vs. 11.0; IQR, 10.0 vs. not acquired; *P* = 0.45).

### Correlations between TLV and MFIS, MFI, and PES in MS patients

**Fig 2:**
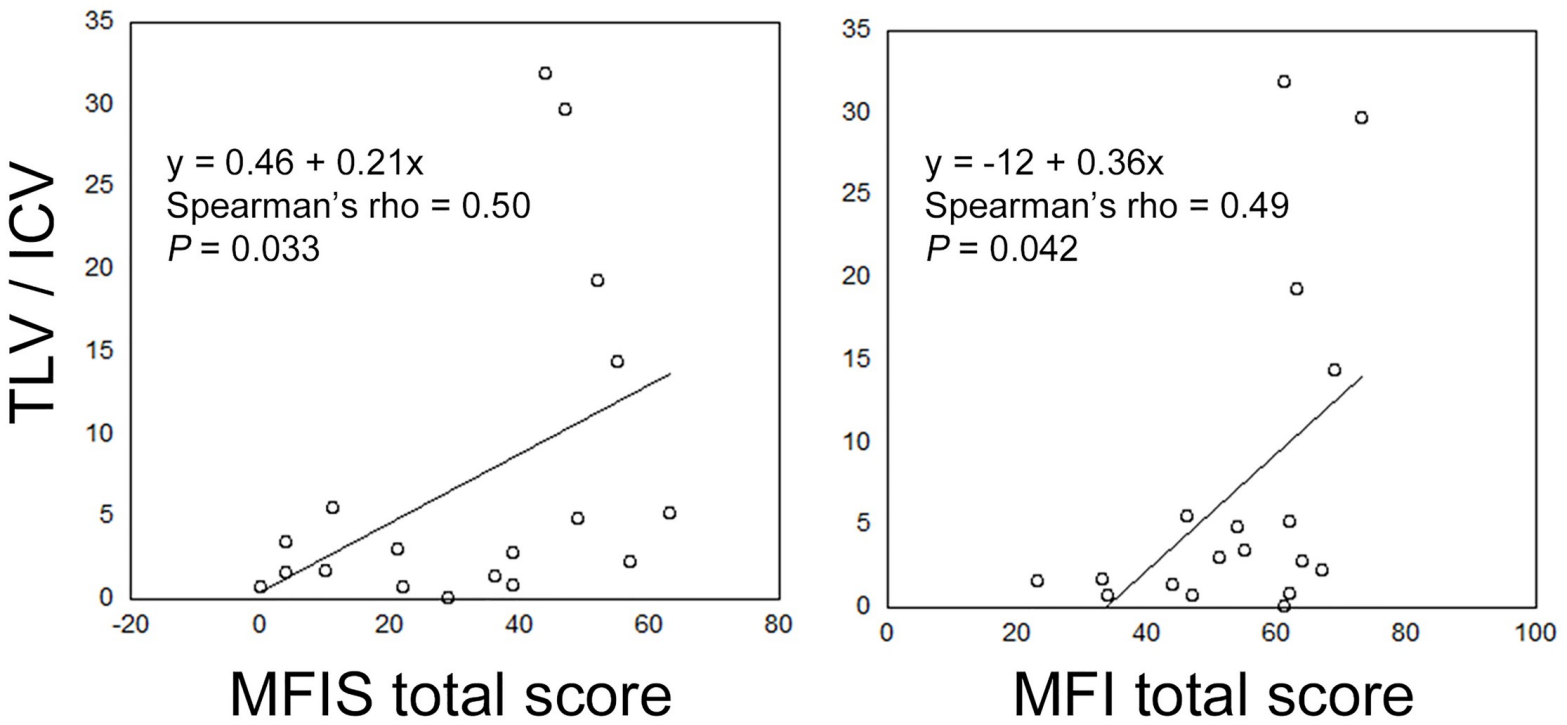
Correlations between TLV/ICV and MFIS total score and between TLV/ICV and MFI total score in patients with MS. ICV, intracranial volume; MFI, Multidimensional Fatigue Inventory; MFIS, Modified Fatigue Impact Scale; MS, multiple sclerosis; TLV, total lesion volume.

As expected, the number of MS patients without brain abnormalities was extremely low (*n* = 2). Since the number in a group is less than five, it is not considered to get a proper result to analyze the data by using Mann-Whitney U test, the correlations between TLV and scores, including MFIS, MFI, and PES, were investigated only in MS patients. Eighteen MS patients whose MRI was performed within 60 days before MFIS were included to the correlation study. TLV/ICV demonstrated a positive correlation with MFIS and MFI (Spearman’s ρ = 0.50 and 0.49, *P* = 0.033 and 0.042, respectively; Fig 2). PES showed no correlation with TLV/ICV (Spearman’s ρ = 0.44, *P* = 0.068). Moreover, the physical subscale in MFIS positively correlated with TLV/ICV (Spearman’s ρ = 0.51, *P* = 0.031), but the cognitive and psychosocial subscale in MFIS showed no correlation with TLV/ICV (*P* > 0.099).

## Discussion

Our previous study showed that MFIS and MFI were higher in NMOSD [20] and MS patients [21] than in normal controls. In this study, the results showed no differences in MFIS and MFI between NMOSD and MS patients. These results are in agreement with those of a previous study by Akaishi et al [9]. However, our results suggest that the cause of fatigue may be different in NMOSD and MS patients.

The PES scores were higher in NMOSD than in MS patients in this study. Several studies reported 75%-90% of patients with MS showed spinal cord lesions [22–25]. The percentages are similar to our study. Although the percentage of patients with a history of myelitis was higher among those with MS than with NMOSD in our study, the PES scores were not different between NMOSD and MS patients with or without a history of myelitis. On the other hand, the percentage of patients with extended spinal cord lesions was significantly higher among those with NMOSD than with MS. Moreover, the PES scores tended to be higher in NMOSD and MS patients with than in those without extended spinal cord lesions. These results suggested that extended spinal cord lesions may have some role in the pathogenesis of pain. PES score positively correlated with MFIS and MFI in NMOSD patients. Therefore, fatigue in NMOSD patients may be associated with pain caused by extended spinal cord lesions. Indeed, other studies reported fatigue were also common in patients with spinal cord injury [26, 27]. However, these reports showed many factors including medication, depression, and pain could affect the fatigue in patients with spinal cord injury. Therefore, further investigation is needed to investigate these relations.

Although the percentage of patients with brain abnormalities was higher in MS than in NMOSD patients, MS patients with or without brain abnormalities showed no differences in MFIS, MFI, and PES. However, the number of MS patients without brain abnormalities was significantly low, so we added the correlation analysis of TLV and MFIS, MFI, and PES. The result showed a positive correlation between TLV and fatigue score, including MFIS and MFI. These results suggest that fatigue in MS patients may be associated with TLV. A previous study reported that high-fatigue MS patients demonstrated significant brain atrophy and lesion volume [28]. These investigators arbitrarily divided MS patients into high- and low-fatigue groups. However, to the best of our knowledge, ours is the first report to show a positive correlation between TLV and fatigue score.

This study has some limitations. First, depression was not investigated in our study. As previously reported, a strong positive correlation was found between depression and fatigue in patients with NMOSD and MS [9]. Another study reported that MS patients with major depression had a greater lesion volume compared to those without depression [29]. Since our results showed a positive correlation between fatigue and TLV in MS patients, fatigue and depression could stem from TLV. Further investigation is required to reveal the relationships among fatigue, depression, and lesion volume. Second, the sleep disorders investigation was not included in our study. Sleep was reported to be related to fatigue in the patients with MS [6, 30–32]. Third, the response rates without any blanks in MFIS/MFI/PES are different between NMOSD and MS patients (66.2% vs 38.5%, respectively). Cognitive impairment or fatigue could lower the response rate, which could be selection bias. Finally, the brain MRI scans were performed using five instruments at random. Although our study obtained results similar to those of a previous study [28], further studies with a higher number of patients and using the same MRI scanner are needed to clarify the correlation between TLV and fatigue.

In conclusion, fatigue was not different between NMOSD and MS patients. On the other hand, patients with NMOSD suffered from more severe pain compared with patients with MS. Brain lesions in MS patients were positively correlated with fatigue, meanwhile patients with the extended spinal cord lesions showed the higher PES score. This study may help to develop more appropriate treatment strategies in patients with NMOSD and MS.

## References

[1] Wingerchuk DM, Banwell B, Bennett JL, Cabre P, Carroll W, Chitnis T, et al International consensus diagnostic criteria for neuromyelitis optica spectrum disorders. Neurology. 2015;85(2):177–89. 10.1212/WNL.0000000000001729 26092914 PMC4515040

[2] Lennon VA, Wingerchuk DM, Kryzer TJ, Pittock SJ, Lucchinetti CF, Fujihara K, et al A serum autoantibody marker of neuromyelitis optica: distinction from multiple sclerosis. Lancet (London, England). 2004;364(9451):2106–12. Epub 2004/12/14. 10.1016/s0140-6736(04)17551-x . 15589308

[3] Geraldes R, Ciccarelli O, Barkhof F, De Stefano N, Enzinger C, Filippi M, et al The current role of MRI in differentiating multiple sclerosis from its imaging mimics. Nat Rev Neurol. 2018;14(4):199–213. Epub 2018/03/10. 10.1038/nrneurol.2018.14 . 29582852

[4] Polman CH, Reingold SC, Banwell B, Clanet M, Cohen JA, Filippi M, et al Diagnostic criteria for multiple sclerosis: 2010 revisions to the McDonald criteria. Annals of neurology. 2011;69(2):292–302. 10.1002/ana.22366 21387374 PMC3084507

[5] Pan J, Zhao P, Cai H, Su L, Wood K, Shi FD, et al Hypoxemia, Sleep Disturbances, and Depression Correlated with Fatigue in Neuromyelitis Optica Spectrum Disorder. CNS neuroscience & therapeutics. 2015;21(7):599–606. Epub 2015/06/03. 10.1111/cns.12411 . 26031911 PMC4478121

[6] Penner IK, Paul F. Fatigue as a symptom or comorbidity of neurological diseases. Nat Rev Neurol. 2017;13(11):662–75. Epub 2017/10/14. 10.1038/nrneurol.2017.117 . 29027539

[7] Kos D, Kerckhofs E, Nagels G, D’Hooghe M B, Ilsbroukx S. Origin of fatigue in multiple sclerosis: review of the literature. Neurorehabilitation and neural repair. 2008;22(1):91–100. Epub 2007/04/06. 10.1177/1545968306298934 . 17409388

[8] Beekman J, Keisler A, Pedraza O, Haramura M, Gianella-Borradori A, Katz E, et al Neuromyelitis optica spectrum disorder: Patient experience and quality of life. Neurology(R) neuroimmunology & neuroinflammation. 2019;6(4):e580 Epub 2019/07/30. 10.1212/nxi.0000000000000580 . 31355316 PMC6624099

[9] Akaishi T, Nakashima I, Misu T, Fujihara K, Aoki M. Depressive state and chronic fatigue in multiple sclerosis and neuromyelitis optica. J Neuroimmunol. 2015;283:70–3. 10.1016/j.jneuroim.2015.05.007 . 26004160

[10] Muto M, Mori M, Sato Y, Uzawa A, Masuda S, Uchida T, et al Current symptomatology in multiple sclerosis and neuromyelitis optica. European journal of neurology. 2015;22(2):299–304. Epub 2014/09/30. 10.1111/ene.12566 . 25264295

[11] Chavarro VS, Mealy MA, Simpson A, Lacheta A, Pache F, Ruprecht K, et al Insufficient treatment of severe depression in neuromyelitis optica spectrum disorder. Neurology(R) neuroimmunology & neuroinflammation. 2016;3(6):e286 Epub 2016/11/02. 10.1212/nxi.0000000000000286 . 27800532 PMC5079380

[12] von Bismarck O, Dankowski T, Ambrosius B, Hessler N, Antony G, Ziegler A, et al Treatment choices and neuropsychological symptoms of a large cohort of early MS. Neurology(R) neuroimmunology & neuroinflammation. 2018;5(3):e446 Epub 2018/03/08. 10.1212/nxi.0000000000000446 . 29511705 PMC5833336

[13] O’Hara L, Cadbury H, De SL, Ide L. Evaluation of the effectiveness of professionally guided self-care for people with multiple sclerosis living in the community: a randomized controlled trial. Clinical rehabilitation. 2002;16(2):119–28. Epub 2002/03/26. 10.1191/0269215502cr478oa . 11911510

[14] Mohr DC, Hart SL, Goldberg A. Effects of treatment for depression on fatigue in multiple sclerosis. Psychosomatic medicine. 2003;65(4):542–7. Epub 2003/07/29. 10.1097/01.psy.0000074757.11682.96 . 12883103

[15] Tackley G, Vecchio D, Hamid S, Jurynczyk M, Kong Y, Gore R, et al Chronic neuropathic pain severity is determined by lesion level in aquaporin 4-antibody-positive myelitis. J Neurol Neurosurg Psychiatry. 2017;88(2):165–9. 10.1136/jnnp-2016-314991 . 27884934

[16] Kanamori Y, Nakashima I, Takai Y, Nishiyama S, Kuroda H, Takahashi T, et al Pain in neuromyelitis optica and its effect on quality of life: a cross-sectional study. Neurology. 2011;77(7):652–8. Epub 2011/08/05. 10.1212/WNL.0b013e318229e694 . 21813781

[17] Marck CH, De Livera AM, Weiland TJ, Jelinek PL, Neate SL, Brown CR, et al Pain in People with Multiple Sclerosis: Associations with Modifiable Lifestyle Factors, Fatigue, Depression, Anxiety, and Mental Health Quality of Life. Frontiers in neurology. 2017;8:461 Epub 2017/09/21. 10.3389/fneur.2017.00461 . 28928713 PMC5591834

[18] Hayakawa S, Mori M, Okuta A, Kamegawa A, Fujiyoshi Y, Yoshiyama Y, et al Neuromyelitis optica and anti-aquaporin-4 antibodies measured by an enzyme-linked immunosorbent assay. J Neuroimmunol. 2008;196(1–2):181–7. Epub 2008/05/09. 10.1016/j.jneuroim.2008.03.009 . 18462810

[19] Schmidt P, Gaser C, Arsic M, Buck D, Förschler A, Berthele A, et al An automated tool for detection of FLAIR-hyperintense white-matter lesions in multiple sclerosis. Neuroimage. 2012;59(4):3774–83. 10.1016/j.neuroimage.2011.11.032 22119648

[20] Masuda H, Mori M, Uzawa A, Muto M, Uchida T, Kobayashi S, et al Validation of the Japanese version of the Modified Fatigue Impact Scale and assessment of the effect of pain on scale responses in patients with multiple sclerosis. Clinical and Experimental Neuroimmunology. 2015;6:409–12.

[21] Masuda H, Mori M, Uzawa A, Uchida T, Ohtani R, Kobayashi S, et al Validation of the Modified Fatigue Impact Scale and the relationships among fatigue, pain and serum interleukin-6 levels in patients with neuromyelitis optica spectrum disorder. Journal of the neurological sciences. 2018;385:64–8. Epub 2018/02/07. 10.1016/j.jns.2017.11.041 . 29406915

[22] Qiu W, Raven S, James I, Luo Y, Wu J, Castley A, et al Spinal cord involvement in multiple sclerosis: a correlative MRI and high-resolution HLA-DRB1 genotyping study. Journal of the neurological sciences. 2011;300(1–2):114–9. Epub 2010/10/05. 10.1016/j.jns.2010.09.006 . 20884011

[23] Nijeholt GJ, van Walderveen MA, Castelijns JA, van Waesberghe JH, Polman C, Scheltens P, et al Brain and spinal cord abnormalities in multiple sclerosis. Correlation between MRI parameters, clinical subtypes and symptoms. Brain. 1998;121 (Pt 4):687–97. Epub 1998/05/13. 10.1093/brain/121.4.687 . 9577394

[24] Bot JC, Barkhof F, Polman CH, Lycklama a Nijeholt GJ, de Groot V, Bergers E, et al Spinal cord abnormalities in recently diagnosed MS patients: added value of spinal MRI examination. Neurology. 2004;62(2):226–33. Epub 2004/01/28. 10.1212/wnl.62.2.226 . 14745058

[25] Galler S, Stellmann JP, Young KL, Kutzner D, Heesen C, Fiehler J, et al Improved Lesion Detection by Using Axial T2-Weighted MRI with Full Spinal Cord Coverage in Multiple Sclerosis. AJNR American journal of neuroradiology. 2016;37(5):963–9. Epub 2016/01/09. 10.3174/ajnr.A4638 . 26744444 PMC7960307

[26] Cudeiro-Blanco J, Onate-Figuerez A, Soto-Leon V, Avendano-Coy J, Mordillo-Mateos L, Brocalero-Camacho A, et al Prevalence of Fatigue and Associated Factors in a Spinal Cord Injury Population: Data from an Internet-Based and Face-to-Face Surveys. Journal of neurotrauma. 2017;34(15):2335–41. Epub 2017/06/02. 10.1089/neu.2016.4950 . 28569601

[27] Lee AK, Miller WC, Townson AF, Anton HA. Medication use is associated with fatigue in a sample of community-living individuals who have a spinal cord injury: a chart review. Spinal cord. 2010;48(5):429–33. Epub 2009/11/18. 10.1038/sc.2009.145 . 19918252

[28] Tedeschi G, Dinacci D, Lavorgna L, Prinster A, Savettieri G, Quattrone A, et al Correlation between fatigue and brain atrophy and lesion load in multiple sclerosis patients independent of disability. Journal of the neurological sciences. 2007;263(1–2):15–9. Epub 2007/08/04. 10.1016/j.jns.2007.07.004 . 17673234

[29] Feinstein A, Roy P, Lobaugh N, Feinstein K, O’Connor P, Black S. Structural brain abnormalities in multiple sclerosis patients with major depression. Neurology. 2004;62(4):586–90. Epub 2004/02/26. 10.1212/01.wnl.0000110316.12086.0c . 14981175

[30] Stanton BR, Barnes F, Silber E. Sleep and fatigue in multiple sclerosis. Mult Scler. 2006;12(4):481–6. Epub 2006/08/12. 10.1191/135248506ms1320oa . 16900762

[31] Veauthier C, Radbruch H, Gaede G, Pfueller CF, Dorr J, Bellmann-Strobl J, et al Fatigue in multiple sclerosis is closely related to sleep disorders: a polysomnographic cross-sectional study. Mult Scler. 2011;17(5):613–22. Epub 2011/02/01. 10.1177/1352458510393772 . 21278050

[32] Veauthier C, Gaede G, Radbruch H, Gottschalk S, Wernecke KD, Paul F. Treatment of sleep disorders may improve fatigue in multiple sclerosis. Clinical neurology and neurosurgery. 2013;115(9):1826–30. Epub 2013/06/15. 10.1016/j.clineuro.2013.05.018 . 23764040

